# Integrated Mobile Platform for Geodetic Fieldwork: Architecture, GNSS Positioning, and Data Management

**DOI:** 10.3390/s26206614

**Published:** 2026-10-19

**Authors:** Wojciech Matwij, Mateusz Jabłoński, Kinga Dudek, Kamil Łazarski, Agata Ostrowska, Kamila Krajewska, Paweł Krajewski

**Affiliations:** AGH University of Krakow, Faculty of Geo-Data Science, Geodesy, and Environmental Engineering, Department of Mining Area Protection, Geoinformatics and Mining Surveying, al. A. Mickiewicza 30, 30-059 Krakow, Poland; mjablonski@agh.edu.pl (M.J.); kingula@student.agh.edu.pl (K.D.); klazarski@student.agh.edu.pl (K.Ł.); agaostrowska@student.agh.edu.pl (A.O.); kkrajewska@student.agh.edu.pl (K.K.); krajewski@student.agh.edu.pl (P.K.)

**Keywords:** mobile GIS, geodetic fieldwork, smartphone positioning, offline-first application, coordinate transformation, geodetic control points

## Abstract

**Highlights:**

**What are the main findings?**

- A modular Android application, “Do Punktu”, was developed to integrate geodetic point management, coordinate transformations, mapping, photographic documentation, data exchange, reporting, and offline fieldwork in a single mobile platform.
- Functional tests confirmed the operation of the main application modules, while preliminary field observations indicated smartphone-based navigation offsets of approximately 4–5 m in open areas and 10 m in forested areas.

**What are the implications of the main findings?**

- The proposed offline-first architecture can support the organization, navigation, documentation, and exchange of geodetic field data without replacing professional GNSS surveying equipment.
- The application provides a basis for further research on smartphone-based positioning, external GNSS receiver integration, collaborative data synchronization, and quantitative evaluation of fieldwork efficiency.

**Abstract:**

This paper presents “Do Punktu”, an offline-first Android application designed to support geodetic fieldwork involving the localization, documentation, and management of geodetic control and project points. The system integrates a local SQLite database, digital maps, mobile-device Global Navigation Satellite System (GNSS) positioning, coordinate transformations, height data, photographic documentation, point navigation, stakeout support, data exchange, Portable Document Format (PDF) reporting, and project backup. The application was implemented in Kotlin using Jetpack Compose UI 1.9.2 and Material 3 1.3.0 and supports World Geodetic System 1984 (WGS84) and the Polish national projected coordinate systems PL-1992 (EPSG:2180) and PL-2000 (EPSG:2176–2179). The project produced an inventory database containing 539 point records. The application was subsequently evaluated through functional testing involving 16 testers and preliminary field testing involving 11 surveyors. Exploratory observations indicated approximate distances of 4–5 m from the target point in open terrain and approximately 10 m in forested areas. These values are reported as preliminary observations rather than as a formal accuracy assessment. The results confirm the feasibility of integrating mobile sensing, geodetic data management, and field documentation in one local application. Further studies should include controlled experiments, external GNSS receivers, quantitative usability assessment, and statistical evaluation of positioning accuracy and point-finding efficiency.

## 1. Introduction and the Functional Gap

Geodetic control points constitute one of the fundamental elements of surveying operations. They are used, among other purposes, in the establishment of construction control networks, topographic and elevation surveys, construction surveying, control measurements, object inventorying, and the monitoring of displacements and deformations. Their importance results from the need to relate detailed measurements to an adopted spatial reference system. The design, optimization, and accuracy assessment of geodetic control networks have been the subject of geodetic research for many years [1,2].

In field practice, one of the first stages of many geodetic tasks is the preparation of a set of reference points and their identification in the field. This process usually involves obtaining information about the points, analyzing their locations, preparing field materials, navigating to the points, assessing their condition, preparing documentation, and, where necessary, recording updated information. Despite the development of surveying and information technologies, these activities are still frequently performed using multiple independent tools, such as field notebooks, field sketches, geoportals, GNSS receivers, text files, mapping applications, photographic documentation, and spreadsheets [3,4].

The development of GNSS technology, mobile devices, and location-based services has significantly changed the way spatial data are acquired, presented, and updated. Modern smartphones and tablets enable users to display data on maps, record their current position, prepare photographic documentation, store data locally, and exchange information with external systems [5,6]. Location-based services are currently used in both private and professional applications. Their further development involves the integration of spatial data, contextual information, and user interaction with the surrounding environment [3].

In geodetic applications, mobile devices can serve as supplementary field tools supporting work organization, point localization, the collection of descriptive information, and the documentation of object conditions [6]. Available solutions, such as geoportals, mapping applications, and mobile geographic information system (GIS) tools, enable spatial data to be accessed directly in the field [4,7]. Their use significantly facilitates access to information; however, these solutions do not always meet the specific requirements of geodetic work involving control points and design points.

One of the important practical problems is the identification of points in the field. Topographic descriptions may be outdated, while points may be obscured by vegetation, damaged, reconstructed, displaced, or difficult to access. Changes in land development, the construction of new facilities, infrastructure renovations, and natural environmental processes mean that information stored in databases does not always correspond to the current field conditions. Consequently, a surveyor needs not only access to the coordinates of a point but also a tool that enables project organization, navigation to the point, documentation of its condition, and subsequent data exchange.

Existing mobile GIS and commercial surveying applications already provide many of the functions required for geodetic fieldwork. The motivation for developing “Do Punktu” was to provide a focused smartphone workflow for locating predefined points, documenting their condition, and managing project information, photographs, and data exchange. The application was designed for surveyors, students, and other field personnel, with an emphasis on ease of use, local data storage, and operation without configuring external surveying instruments. Its coordinate and height reference support and Geoportal basemaps were selected for the Polish geodetic context.

In response to this need, the “Do Punktu” mobile application was developed to support field geodetic work related to the management, identification, documentation, and exchange of data concerning geodetic points. The application was designed for the Android operating system and combines a local database of projects and points, basemap support, data import and export, coordinate transformations, height data management, photographic documentation, GNSS measurements, and point stakeout functionality.

The aim of this study is to present the design and implementation of the “Do Punktu” application as a mobile platform supporting field surveying activities. The article discusses the design assumptions, system architecture, data model, technologies used, and the main functionalities of the application. Particular attention is given to the integration of typically geodetic functions with mechanisms characteristic of mobile applications, including maps, GNSS-based positioning, photographic documentation, and local data storage [3,8].

The main contribution of this study is threefold. First, it presents an offline-first mobile architecture tailored to geodetic fieldwork with point-based data, integrating project management, local storage, coordinate and height reference system support, mapping, photographic documentation, data exchange, and reporting within a single Android application. Second, it describes a geodetic data model that links project, point, height, status, and documentation information in a form suitable for field use and later exchange. Third, it reports functional and preliminary field testing of the implemented system using a 539-point inventory database and a group of field users. The contribution is therefore primarily architectural and implementation-oriented, while the GNSS positioning and local correction procedures are treated as supporting and experimental functionalities requiring further quantitative validation.

This work is design- and implementation-oriented. It does not constitute a comprehensive field validation of the accuracy or operational effectiveness of the application. An assessment of the impact of the implemented functions on point identification time, positioning accuracy, and surveyor productivity should constitute a subsequent stage of the research. Nevertheless, the presented system may provide a basis for the further development of specialized mobile tools for geodesy and for future studies on the use of smartphones and external GNSS receivers in field surveying operations.

### Functional Comparison of Existing Solutions

To establish the position of the “Do Punktu” application in relation to existing tools, selected functionalities available in solutions used, or potentially applicable, during fieldwork involving geodetic points were compared (Table 1). The comparison includes geoportals and public administration systems, universal mobile GIS tools, the Metrica application—based on the authors’ assessment of the available publications—and the developed “Do Punktu” application.

Existing spatial data dissemination systems are often designed primarily as tools for accessing reference information. Geoportals and public administration services provide access to geodetic and cartographic resource data, enable data browsing, and allow points to be located on a map; however, they do not always support the complete fieldwork process [7]. In contrast, universal GIS applications, such as QField, offer extensive capabilities for working with spatial layers and GIS projects. Nevertheless, they require prior configuration and are not directly focused on typical workflows involving geodetic control points [4,9].

Commercial Android surveying applications, including X-Survey, SurPAD, FieldGenius, and Carlson Layout, already provide overlapping capabilities for point management, coordinate handling, surveying, and stakeout [10,11,12,13]. These products primarily address professional surveying or construction workflows and commonly support external GNSS receivers and, depending on the product, total stations. Their use is not necessarily restricted to external instruments; for example, FieldGenius also supports the internal GNSS receiver of an Android device [12].

“Do Punktu” was designed with a narrower operational focus: helping users locate predefined points, record their condition, attach photographs and descriptions, and manage the resulting documentation on an ordinary smartphone. Its intended users include surveyors, students, and other field personnel who need these supporting functions without configuring a professional surveying instrument. Ease of use and reduced reliance on specialist configuration are design objectives; comparative usability advantages over commercial applications have not been established in the present study.

The thematically closest solution described in the literature is the Metrica application. Publications concerning this system present its motivation, design assumptions, practical verification, and architecture as a low-cost mobile application supporting the collection of information about geodetic control points and navigation to those points [8,14,15]. These studies confirm the validity of developing specialized mobile tools for surveyors and demonstrate that the design of such applications can be analyzed not only from a usability perspective but also as a problem involving system architecture, data organization, and information flow.

The publication concerning the architecture of the Metrica system constitutes an important reference point for the present study. The authors demonstrated that available mobile technologies can be used to develop a specialized tool supporting surveyors in their work with control points [8]. However, that study focused primarily on system architecture, scalability, the community-based model, and the possibility of user-driven data updates rather than on the integration of a broader set of geodetic functions within a local field application. The available publications describe Metrica as a design- and prototype-oriented solution developed as part of diploma projects and research on the architecture of low-cost location-based applications for professional communities. No publicly available, currently maintained version of the application or open-source code repository enabling independent reproduction and direct functional comparison of the system was identified. Therefore, in this study, Metrica is treated as an important literary and conceptual reference rather than as a tool subjected to an operational comparison with the “Do Punktu” application.

**Table 1:** Comparison of selected functionalities of the geoportal, QField, the Metrica application, and the “Do Punktu” application. Source: authors’ elaboration based on documentation and publications [7,8,9,14,15].

Functionality	Geoportal	QField/GIS	Metrica	Do Punktu
Displaying points on a map	Yes	Yes	Yes	Yes
Access to reference data	Yes	Dependent	Yes	Dependent on user import
Local project database	No	Yes	Limited/System-dependent	Yes
Support for multiple field projects	Limited	Yes	No unambiguous confirmation available	Yes
Adding user-defined points	Limited	Yes	Yes	Yes
Editing point information	Limited	Yes	Yes	Yes
Photographic documentation of points	No/Limited	Yes	Yes	Yes
Attachments associated with points	No/Limited	Yes	Partially	Yes
Navigation to a point	Limited	Dependent	Yes, via Google Maps	Yes, within the app
GNSS position measurement within the application	No	Dependent	Identified as a direction for future development	Yes
Stakeout of points	No	Dependent	No	Yes
Support for the PL-1992 coordinate system	Yes	Dependent	No	Yes
Support for the PL-2000 coordinate system	Yes	Dependent	No	Yes
Support for WGS84	Yes	Yes	Yes	Yes
Support for vertical reference systems	Yes/Informational only	Dependent	No	Yes
Coordinate transformations	External services	Dependent	No	Yes
CSV import	No/Limited	Yes	No	Yes
CSV export	Limited	Yes	No	Yes
KML import/export	Limited	Yes	No	Yes
PDF reports for points	No	Dependent	No	Yes
Data backup	Not applicable	Dependent	No	Yes
Offline operation	Limited	Yes	Identified as a requirement	Yes, for core local functions
Online updating by project participants	No	Dependent	Planned	Planned
Publicly available application for testing	Yes	Yes	No	Research prototype

The comparison uses the following qualitative categories. “Yes” indicates that the function is supported according to the documentation or publications reviewed. “No” indicates that the function is not supported within the described scope. “Dependent” indicates that availability depends on project configuration, an external service, or additional resources. “Limited” indicates that the function is available only within a restricted scope or under specific conditions. “Partially” indicates that only some components of the listed function are supported. “Planned” denotes a function identified as a future development direction. Where the available sources do not allow a clear assessment, this is stated explicitly. The table summarizes documented functionality and does not represent a comparative performance evaluation.

The comparative analysis indicates that geoportals and public administration services primarily serve as sources of reference data [7]. Universal GIS applications provide considerable flexibility and extensive configuration options; however, they are not directly focused on workflows involving geodetic control points [4]. Metrica represents the closest reference in the literature because it addresses control points and navigation to these points. Nevertheless, the available publications describe it mainly as a prototype and an example of a location-based application architecture, which prevents a complete functional comparison and the use or verification of its source code [8]. Based on an analysis of the cited publications and publicly available materials, neither a current version of the application nor access to its repository could be confirmed. Against this background, the “Do Punktu” application was designed as a local, integrated mobile environment combining organizational, geodetic, and documentation-related functionalities.

## 2. Design Assumptions of the “Do Punktu” Application 

The design of the “Do Punktu” application was based on the assumption that a mobile device can serve as an integrated tool supporting the fieldwork stage of a surveyor’s activities. Unlike solutions intended solely for browsing spatial data or navigating to a specified location, the proposed application was designed to enable the management of geodetic points within separate projects, operation with a local database, support for the basic coordinate systems used in Poland, documentation of point conditions, and data exchange with other systems [16].

The primary design objective was to combine ease of use with the functionality required during fieldwork [17,18]. The application was intended to be usable without specialized training while providing functions relevant to surveyors, including coordinate transformations, height-system support, data import and export, GNSS positioning, point stakeout, and the generation of documentation. It was also assumed that the application should operate on standard Android mobile devices, without requiring additional equipment in its basic operating mode.

### 2.1. Functional Requirements

The functional requirements were defined on the basis of a typical workflow for field surveying activities involving geodetic control points and design points. They include data preparation, project organization, map-based work, locating points, documentation, and the export of results.

The main functional requirements included:

- creating and managing multiple geodetic projects;
- storing points in a local database;
- manually adding, editing, and deleting points;
- importing points from files used in surveying practice;
- exporting data to formats suitable for further use;
- displaying points on base maps;
- supporting OpenStreetMap and Geoportal orthophoto maps;
- supporting coordinates in the WGS84, PL-1992, and PL-2000 coordinate systems;
- supporting GNSS-derived heights and selected vertical reference systems;
- creating photographic documentation of points;
- supporting attachments;
- measuring the current GNSS position of the device;
- guiding the user to a point;
- staking out points based on their coordinates;
- generating PDF reports;
- creating backups of project data.

An important assumption was that the application should retain a local operating model. Project and point data should be stored on the user’s device, thereby reducing dependence on Internet access. Network connectivity is primarily required when using base maps or external services, whereas the project and point database can be operated locally.

### 2.2. Non-Functional Requirements

In addition to the functional requirements, non-functional requirements were defined concerning usability, reliability, and the possibility of further application development. During fieldwork, particular importance is attached to operational speed, interface simplicity, and the ability to use the application under varying environmental conditions [18,19]. The interface should be clear, and the most important functions should be accessible without requiring the user to navigate through complex menu structures.

The main non-functional requirements included:

- a simple and clear user interface;
- the ability to operate the application in field conditions;
- minimizing the number of actions required to locate a point;
- local data storage;
- resilience to temporary loss of Internet connectivity;
- support for further modular expansion;
- the use of publicly available libraries and technologies [20,21,22,23];
- the possibility of integrating external data sources;
- the possibility of future integration with external GNSS receivers;
- preparation of the application for further field validation.

From a design perspective, limiting dependence on a single data source was also important. The application should allow users to work both with manually entered points and with points imported from files. This approach makes it possible to use the application not only for geodetic control points but also for design, control, surveying, or other user-defined points.

### 2.3. Scope of the First System Version

The first version of the application was designed as a research-and-development prototype intended to verify the feasibility of integrating the most important fieldwork functions within a single mobile tool. Accordingly, the scope of the first version includes all functional requirements described in Section 2.1.

The current implementation uses location updates supplied by Android on a smartphone and does not directly communicate with external surveying GNSS receivers. Positioning is intended to support approximate navigation and point identification. No fixed positioning accuracy is specified or guaranteed, because performance depends on the device, positioning provider, satellite visibility, multipath, and other observation conditions. The application does not process raw GNSS observations to compute an independent positioning solution. The use of raw GNSS observations recorded by Android devices for positioning has been reviewed by Skorupa [24]; this approach is outside the scope of the present implementation. It uses the positions delivered by the operating system, with optional averaging and the experimental local correction described in Section 4.6. The observed navigation offsets reported in Section 5.4 do not establish a general accuracy specification. Integration with external GNSS receivers and controlled evaluation of positioning errors remain future development and research tasks.

This scope enables the “Do Punktu” application to be treated as a development platform. The implemented architecture supports further system extensions involving functions that require more advanced testing, such as integration with RTK receivers, support for external GNSS antennas, data synchronization, a shared point database, automatic assessment of point condition, and analysis of the effectiveness of locating points under different field conditions.

## 3. System Architecture

The “Do Punktu” application was implemented as a native mobile application for the Android operating system [20]. It was developed using the Kotlin programming language and a user interface based on Jetpack Compose UI 1.9.2 and Material 3 1.3.0.. The system also incorporates a local SQLite database, the osmdroid library for map support, and the Proj4J library for coordinate transformations [20,21,22,23].

The adopted architecture is local by design, meaning that the primary project and point data are stored directly on the user’s mobile device [21]. This approach reduces dependence on an Internet connection and enables basic project data to be accessed and managed without continuous network availability, including under conditions of limited connectivity [6,8].

The system was designed as a set of cooperating functional modules. These include:

- the user-interface layer;
- the project and point management layer;
- the local database;
- the map module;
- the coordinate-transformation module;
- the height-transformation module;
- the GNSS module;
- the photographic-documentation module;
- the import and export modules; and
- the reporting module.

This division corresponds to the typical workflow of a surveyor conducting fieldwork: project preparation, entering or importing points, displaying them on a map, locating or staking out a point, documenting its condition, and saving or exporting the results.

### 3.1. General Application Workflow

**Figure 1:**
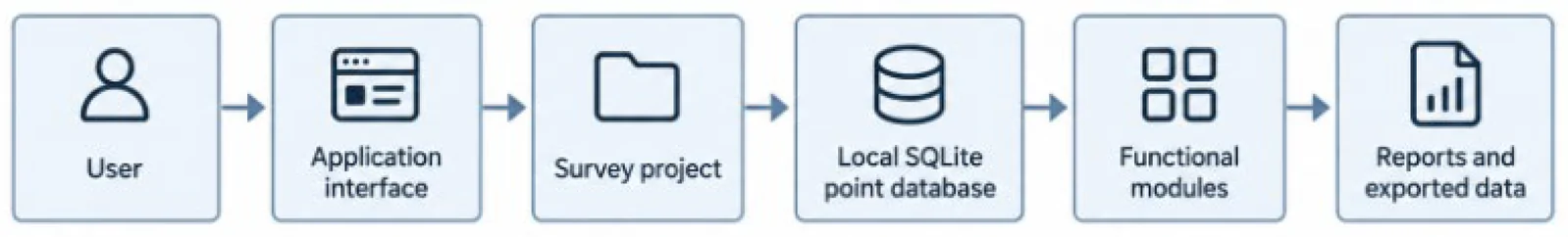
General workflow of the “Do Punktu” mobile application.

The overall data flow within the application begins with the creation of a geodetic project (Figure 1). A project contains a set of points for which coordinates, elevations, status information, descriptions, and paths to photographic documentation are stored. Points can be entered manually, imported from files, or created based on the current GNSS position of the device.

The data are stored in the local SQLite database and can additionally be exported to data-exchange files. This model allows project information to be maintained locally while still supporting interoperability with external software and surveying workflows.

In simplified form, the application’s data flow can be represented as follows:

### 3.2. User-Interface Layer

The user interface was developed using Jetpack Compose [20]. The declarative approach adopted for interface development enables application screens to be implemented as functions describing the current state of the view. The application includes several main screens corresponding to the basic stages of fieldwork:

- the start screen;
- the map screen;
- the point list;
- point details;
- point creation;
- stakeout and navigation;
- project management;
- data import and export;
- reports;
- coordinate transformation; and
- settings.

The user-interface layer acts as an intermediary between the user and the data and computational modules. Users can perform operations on projects and points, select a coordinate reference system, launch the map, add photographic documentation, initiate GNSS positioning, generate reports, and export data. Individual application screens were designed to minimize the number of actions required to perform basic field operations [18,19].

The primary component of the data layer is the local SQLite database, which stores projects, points, and associations between points and their documentation [21]. The detailed data model is presented in Section 4; therefore, this section identifies only its main elements from the perspective of system architecture:

- project data;
- geodetic-point data; and
- attachment data.

Within the data layer, a project functions as a container for points; a point is the basic spatial unit of the application, and photographs and other attachments are associated with specific points. This division separates the organizational structure of a project from the data describing individual points. The use of a local database enables projects to be managed without continuous network access, while data export to exchange files allows the information to be used outside the application.

The central data object of the application is the geodetic point, which includes identification, spatial, height-related, descriptive, and documentation-related information. This section presents only the role of the point within the system architecture; its detailed structure is discussed in Section 4.

### 3.3. Modular System Structure

The map module is responsible for displaying points, the user’s current position, and selected map layers. The application uses the osmdroid library, OpenStreetMap base maps, and the Geoportal orthophoto service provided by the Head Office of Geodesy and Cartography through Web Map Tile Service (WMTS) services [7,22]. In the application architecture, the map module integrates the local point database with the GNSS and coordinate-transformation modules. This enables the visualization of points and the activation of navigation and stakeout functions.

The application supports coordinates in the WGS84, PL-1992 (EPSG:2180), and PL-2000 (EPSG:2176–2179) coordinate reference systems [25]. Coordinate transformations are performed using the Proj4J library and supplementary procedures implemented within the application [23,25]. The transformation module enables points to be converted to WGS84 for map visualization and GNSS positions to be converted to the selected project coordinate system [23,25]. This functionality allows users to work with data stored in coordinate systems commonly used in surveying practice while displaying those data on maps that require geographic coordinates.

The height module supports GNSS ellipsoidal heights and heights in selected vertical reference systems used in Poland. The application is designed to use local height models stored as grid files. By interpolating values from a height-model grid, basic height transformations can be performed locally, without the need to access external services for every calculation.

The GNSS module uses location data provided by the mobile device. The GNSS position can be used to display the user’s current location, create points, navigate to a point, and perform point stakeout. In the basic operating mode, this positioning is intended as a supplementary function and does not replace surveying performed with a professional GNSS receiver [26]. The point-navigation function uses the user’s position and the coordinates of the selected point to determine the distance and direction to the target.

The application enables photographs to be taken and assigned to individual points. The photographs are stored as files in the device’s memory, whereas the database stores paths to those files. This approach reduces the size of the SQLite database and simplifies attachment management. The attachment module is integrated with the reporting and export modules. Consequently, photographs can form part of the project documentation or backup copy, while information about each photograph remains associated with the relevant point.

One of the basic design assumptions was the ability to exchange data with other tools. For this purpose, data import and export were implemented in Comma-Separated Values (CSV), JavaScript Object Notation (JSON), and Keyhole Markup Language (KML)formats. The application also supports the creation of project backups in ZIP (compressed archive) format and the generation of PDF reports. These functions support data archiving and the preparation of documentation related to the inventory of geodetic networks.

The current version of the application is a research-and-development prototype. Its architecture demonstrates the feasibility of integrating multiple geodetic functions within a single mobile tool while providing a basis for further code organization, separation of the business-logic layers, and expansion of external services. Further development directions, including integration with external GNSS receivers, data synchronization, and field validation, are discussed in Section 7.

## 4. Data Model and Implemented Application Functions 

The main organizational unit is the geodetic project, within which a set of points is stored. Each point contains identification information, coordinates, elevations, field status, a description, and associated photographic documentation. Project and point data are stored locally in an SQLite database, whereas photographic files and selected project files are saved in the device’s storage [21].

Point data do not depend on an active connection to an external server, which improves the reliability of field operations. At the same time, the use of data-exchange formats such as CSV, JSON, and KML enables the information to be transferred to other systems at a later stage [27,28,29].

### 4.1. Relationships Between Data Entities

**Figure 2:**
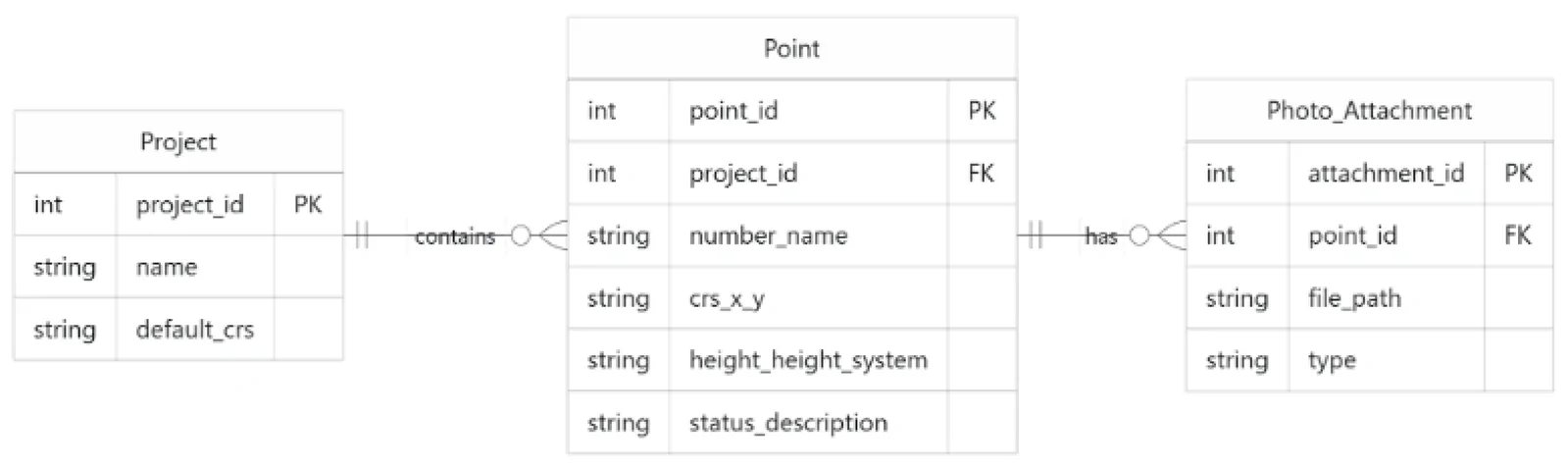
Simplified data model of the “Do Punktu” application, showing the relationships between a project, a geodetic point, and photographic documentation.

The application’s data model has a hierarchical structure (Figure 2). A project can contain multiple points, while each point can have multiple photographs. These relationships can be represented using the following simplified model:

In the SQLite database, the relationship between a project and its points is implemented through a project identifier assigned to each point. The relationship between a point and its photographs is implemented through a separate table containing paths to the image files. Consequently, deleting a project or point can be associated with the deletion of the corresponding dependent data.

In addition to storing data in the SQLite database, the application also generates JSON files describing the project structure. The JSON format makes it possible to store a project, together with a list of points and their attributes, in a form that can be read by other software tools [29]. It can be used for data backup, information exchange, or subsequent analysis of the project structure.

The JSON representation includes, among other elements, the project identifier, name, default coordinate reference system, description, and list of points. For each point, the file stores identification data, coordinates, elevations, status, description, and a list of paths to the associated photographs. This makes it possible to reconstruct the project structure independently of the SQLite database. The use of JSON is also important from the perspective of the application’s further development. This format can support data synchronization with a server, backup creation, and the exchange of projects between users.

The adopted data model allows the “Do Punktu” application to function not only as a mapping tool but also as a local field database for geodetic projects. Point data are stored in a way that enables subsequent editing, transformations, export, reporting, and documentation. The integration of spatial, elevation, descriptive, and photographic information within a single model provides a foundation for the further development of the application.

The most important characteristic of the model is its extensibility. Additional fields can be added to the existing structure, including the measurement date, GNSS position accuracy, number of observations, data source, operator identifier, coordinate-acquisition method, or information concerning synchronization with an external database. Consequently, the current model provides a basis for further research on the assessment of field-data quality and the effectiveness of locating geodetic points.

### 4.2. The Geodetic Project as the Basic Organizational Unit

A geodetic project serves as a container for field data (Table 2). The user can create separate projects for different tasks, areas, or stages of work. Each project has an identifier, name, description, and default coordinate reference system. A list of points is assigned to each project. These points may be entered manually, imported from a file, or created based on the device’s current GNSS position.

This approach makes it possible to separate data originating from different field surveys. This is particularly important in geodetic practice, where a user may simultaneously perform several independent tasks, such as locating control points, inspecting survey points, preparing stakeout operations, or documenting points used for monitoring.

### 4.3. Geodetic Point Model

The central element of the data model is the geodetic point. A point represents a spatial object used in a field project. It may be a geodetic control point, design point, check point, survey point, or any other point defined by the user (Table 3).

From a geodetic perspective, it is important that a point is not represented solely by a pair of horizontal coordinates. The model also supports elevations and information about the vertical reference system. This makes it possible to store data obtained from different sources, such as normal heights in the national vertical reference system or ellipsoidal heights obtained from GNSS measurements.

**Figure 3:**
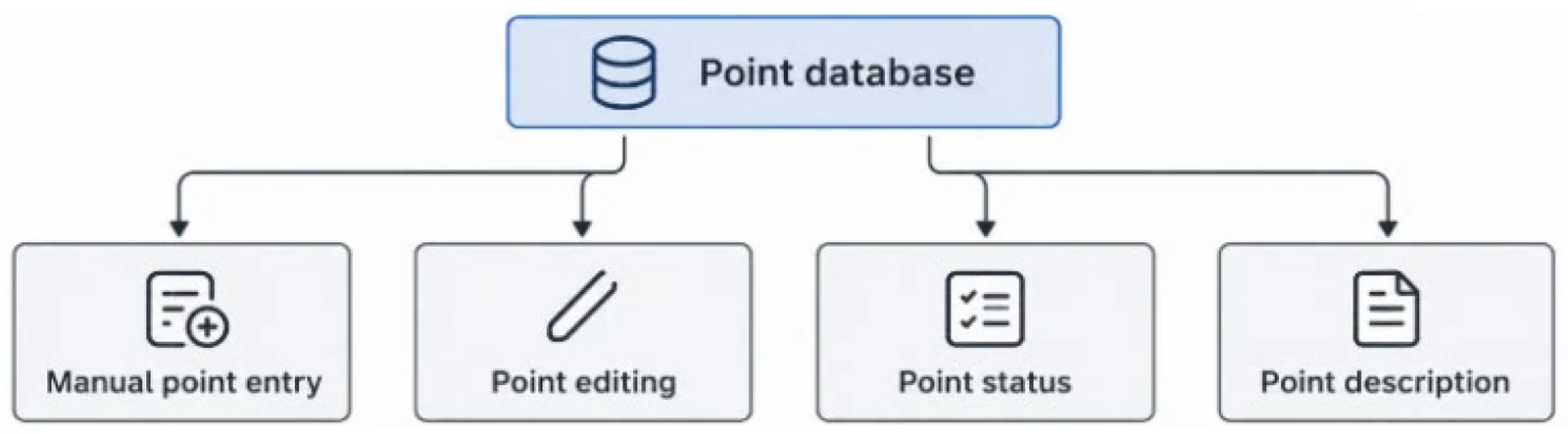
Main components of the point database module implemented in the “Do Punktu” application.

A second core component is the local point database (Figure 3). A point can be added manually, imported from a file, or created based on the device’s current GNSS position. Each point has a number, name, coordinate reference system, horizontal coordinates, elevation, status, description, and optional photographic documentation. The point database module enables users to edit existing points, change their status, and complete descriptive information.

An important feature of the application is the local storage of points. Users are not dependent on continuous access to an external server, which improves the reliability of field operations. The data can subsequently be exported to exchange files or used to prepare a report.

In addition to coordinates and elevations, the application stores information about the status of each point. The status describes the current field condition of the point and can be used when documenting geodetic control points or user-defined design points. The application provides the following basic status categories:

- valid;
- requires verification;
- damaged;
- destroyed;
- not found;
- unknown.

The point status enables the rapid classification of fieldwork results. A surveyor can mark a point as located and valid, indicate the need for additional verification, or specify that the point was not found. This information can later be used to prepare reports, plan subsequent fieldwork, or update a local database.

Photographic documentation can also be associated with each point. Photographs are not stored directly in the database; instead, they are saved as files in the device’s storage. The database stores the paths to these files. This solution reduces the size of the SQLite database and facilitates the management of attachments.

**Figure 4:**
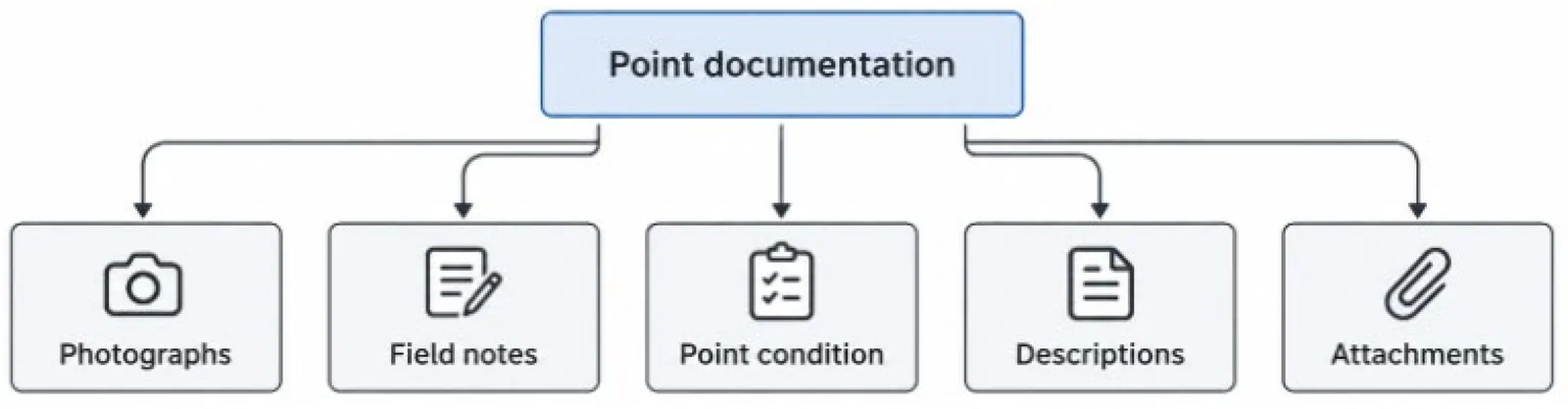
Photographic and descriptive documentation module of the “Do Punktu” application.

Photographic documentation may show the point monument, its surroundings, damage, terrain obstacles, or features that may facilitate its subsequent recovery. Descriptive data complement field information that cannot be derived directly from the coordinates. The documentation function is particularly important for geodetic control points, whose condition may change over time. The ability to store photographs and point status information makes it possible to create a local field-information database. Such a database can subsequently be used during further surveys, for report preparation, or for planning repeated inspections of control points (Figure 4).

### 4.4. Coordinate and Height Reference System Support

The application supports WGS84, PL-1992 (EPSG:2180), and PL-2000 (EPSG:2176–2179) coordinate reference systems [25]. Each point stores its coordinate reference system designation, allowing data from different sources to be managed within one project. The coordinate-transformation module converts point coordinates to WGS84 for map display and smartphone positions to the selected project coordinate reference system [23,25].

The application stores ellipsoidal heights and normal heights referred to the PL-EVRF2007-NH or PL-KRON86-NH height reference frame [25,30,31]. These represent two height types, with normal heights supported in two reference frames. Each point retains its original height value and reference designation, together with any derived height values (Table 4).

Height support includes model-based calculations in addition to metadata storage. The application can download or import the PL-geoid2021 quasi-geoid model and use a locally stored model grid to convert ellipsoidal heights to normal heights in PL-EVRF2007-NH by subtracting the interpolated height anomaly. Conversion between PL-EVRF2007-NH and PL-KRON86-NH uses a separate height-difference grid. The required models must be available locally and cover the point location; if the necessary model value is unavailable, the corresponding derived height is not calculated. The original height and auxiliary model values are retained to distinguish input data from calculated results.

The extended representation of height information is particularly important for the further development of the application. It enables the implementation of height-transformation procedures, verification of data consistency, and preparation for integration with more accurate sources of elevation data.

### 4.5. Mapping Module and Navigation to a Point

The application enables points to be displayed on a map together with the user’s current position. OpenStreetMap is used as the primary map layer, while support is also provided for Geoportal orthophotos delivered through a WMTS service [7,22]. Consequently, the user can work with both a conventional topographic and transportation-oriented basemap and an orthophotographic layer, which can often facilitate the identification of terrain features.

The map serves as the primary field-view interface. The user can observe the position of points relative to their current location, select individual points, open their detailed records, and activate navigation functions. The point-navigation function provides auxiliary support when locating geodetic control points or design points, particularly when the point monument is difficult to identify in the field.

**Figure 5:**
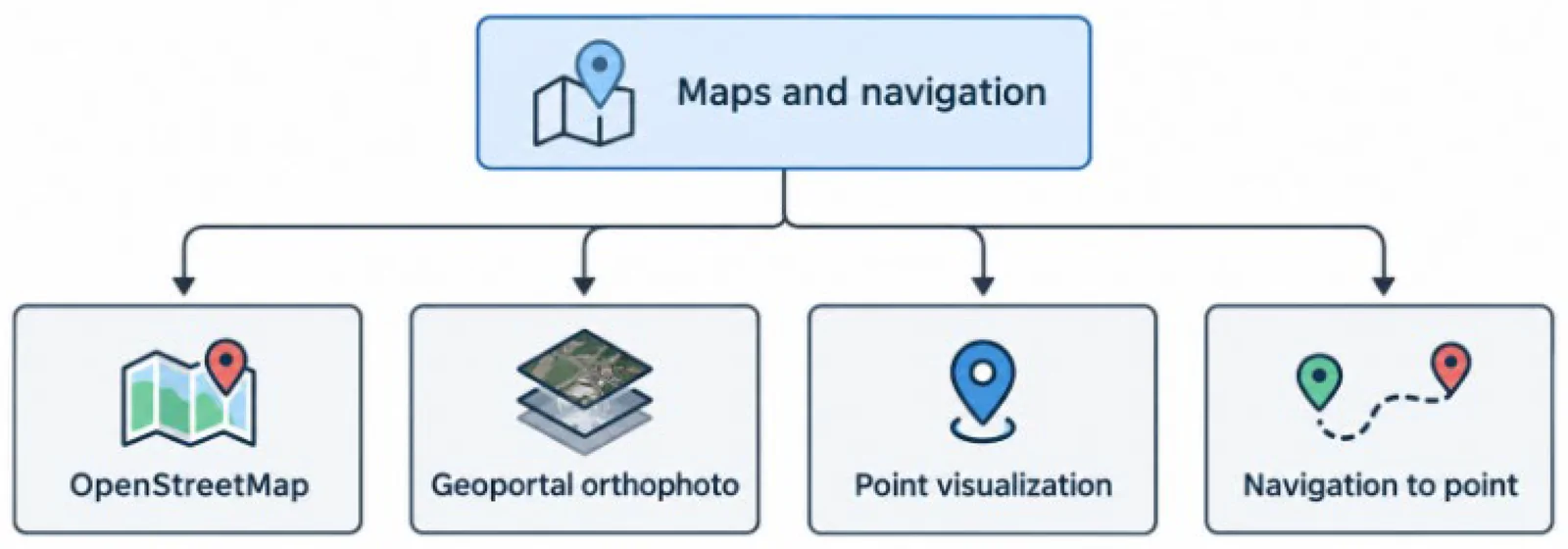
Main components of the maps and navigation module implemented in the “Do Punktu” application.

The use of the map in the application is not limited to visualization. The mapping module integrates data from the local point database, the GNSS module, and the coordinate-transformation module (Figure 5). Points stored in projected coordinate systems must be transformed into a reference system suitable for map display. At the same time, the user’s GNSS-derived position can be used to determine the distance and direction to a selected point [23,25].

The map displays the points stored in the current project and the user’s current position. This improves field orientation and facilitates assessment of the user’s location relative to design or control points. If a GNSS correction has been determined for the current session, the application also displays its status using a symbol with a variable color. This allows the user to quickly determine whether the current position is being displayed with the correction applied and to identify its current status.

### 4.6. GNSS Functions and Experimental Local Position Correction

The application implements an experimental local position-correction procedure based on reference points with known coordinates. Reference coordinates are transformed to WGS84. At each calibration point, location updates are collected for 60 s, and only updates with an available Android-reported horizontal accuracy below 5 m are accepted. These are positions supplied through the Android location interface, which can receive updates from both GPS and network providers.

For reference point i, let φ_i_ and λ_i_ denote the known latitude and longitude, and φ_i_ⱼ and λ_i_ⱼ the coordinates of accepted update j. The calibration position is calculated as a weighted average using Equations (1) and (2):

(1) $$\bar{\varphi_{i}}\;=\;\frac{\sum_{j}v_{\mathrm{ij}}\varphi_{\mathrm{ij}}}{\sum_{j}v_{\mathrm{ij}}},\;\bar{\lambda_{i}}\;=\;\frac{\sum_{j}v_{\mathrm{ij}}\lambda_{\mathrm{ij}}}{\sum_{j}v_{\mathrm{ij}}}$$

(2) $$v_{\mathrm{ij}}=\frac{1}{{\max(a_{\mathrm{ij}},1\;m)}^{2}}$$

Here, a_i_ⱼ is the Android-reported horizontal accuracy. The correction components are the differences between the reference coordinates and the averaged measured coordinates, according to Equation (3):

(3) $${\Delta \varphi }_{i}=\varphi_{i}-\bar{\varphi_{i}},\;{\Delta \lambda }_{i}=\lambda_{i}-\bar{\lambda_{i}}$$

An optional height correction is calculated as Δh_i_ = h_i_ − $\bar{h_{i}}$ when both reference and averaged measured ellipsoidal heights are available. Heights are averaged with the same sample weights, using only updates that contain altitude information.

For a current position, corrections from multiple calibration points are are calculated using Equation (4), taking into account distance, calibration age, and an application-derived accuracy indicator:

(4) $$w_{i}\;=\;\frac{1}{{\max(d_{i},\;5\;m)}^{2}\;\times \;{\left(1\;+\;\frac{t_{i}}{20\;\min}\right)}^{2}\;\times \;{\max(A_{i},\;1\;m)}^{2}},\;A_{i}\;=\;\sqrt{\frac{1}{\sum_{j}v_{\mathrm{ij}}}}$$

The distance d_i_ is measured from the current position to reference point i. The non-negative age t_i_ is calculated from the calibration timestamp and the time stored when the correction-model instance is created. A_i_ is a weighting indicator calculated from the accepted sample accuracies; it is not an independently validated estimate of positioning error. The resulting corrections are then combined using Equation (5):

(5) $$\varphi_{\mathrm{corr}}\;=\;\varphi \;+\;\frac{\sum_{i}w_{i}{\Delta \varphi }_{i}}{\sum_{i}w_{i}},\;\lambda_{\mathrm{corr}}\;=\;\lambda \;+\;\frac{\sum_{i}w_{i}{\Delta \lambda }_{i}}{\sum_{i}w_{i}}$$

The height correction is combined analogously, using only calibration points with an available height correction, and is applied only when the current position contains altitude information. With one calibration point, normalization of the weights gives its full correction. Distance and age therefore change the relative contributions of multiple points rather than progressively reducing a single-point correction to zero.

The implementation provides heuristic distance, age, and quality indicators. A favourable status requires the minimum calibration age to be no more than 20 min and the minimum reference-point distance to be no more than 500 m, with no quality warning. A broader status criterion uses 60 min and 2000 m. The minimum age and minimum distance are evaluated separately and may refer to different calibration points. These status indicators do not automatically exclude older or more distant points from the weighted correction and should not be interpreted as validated spatial or temporal limits.

Correction is suppressed when a severe quality mismatch is detected relative to the nearest calibration point: the current reported horizontal accuracy exceeds 30 m; it exceeds both three times the calibration accuracy indicator and that indicator plus 12 m; or, when satellite-count information is available, the count decreases by at least four. These thresholds are implementation heuristics.

The local correction is an implemented experimental functionality. Its effectiveness was not quantitatively evaluated through a controlled comparison of positioning errors before and after correction in the present study. Its weighting parameters, quality thresholds, and spatial and temporal applicability require further validation.

### 4.7. Import, Export, Backup, and Reporting 

**Figure 6:**
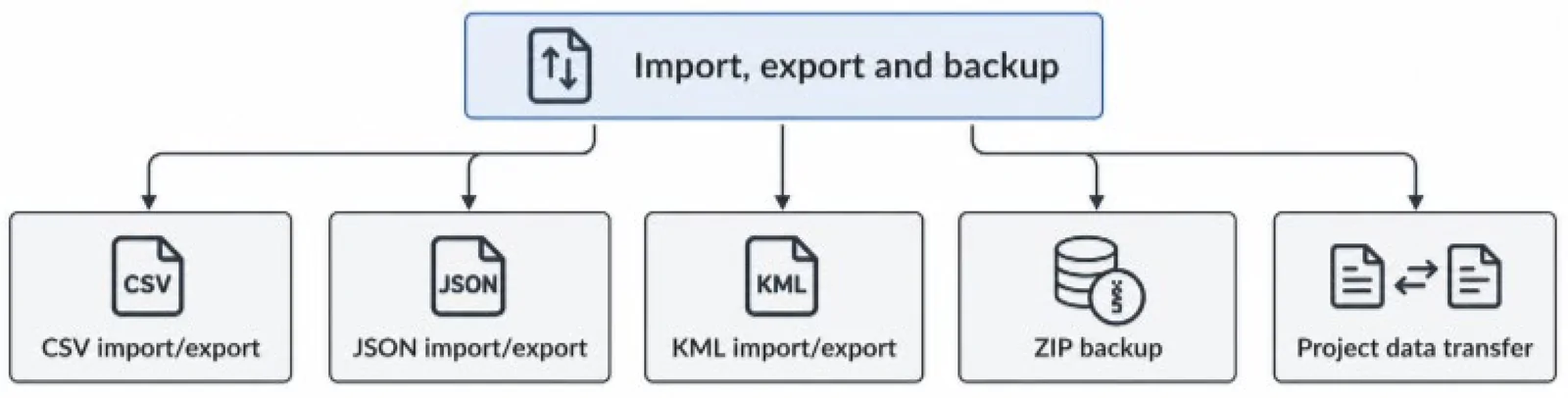
Data exchange and backup module of the “Do Punktu” application.

The application enables data exchange with other tools through support for several file formats (Figure 6). Data import and export have been implemented for CSV, JSON, and KML files [27,28,29]. CSV is useful for exchanging tabular data, JSON enables the storage of a more comprehensive project structure, while KML allows points to be used in tools that support geographic data [27,28,29]. In addition, the application supports the creation of ZIP backups. A backup may include project data and associated files, facilitating data archiving or transfer between devices. This function is of considerable practical importance because field data collected on a mobile device should be capable of being securely preserved and restored.

**Figure 7:**
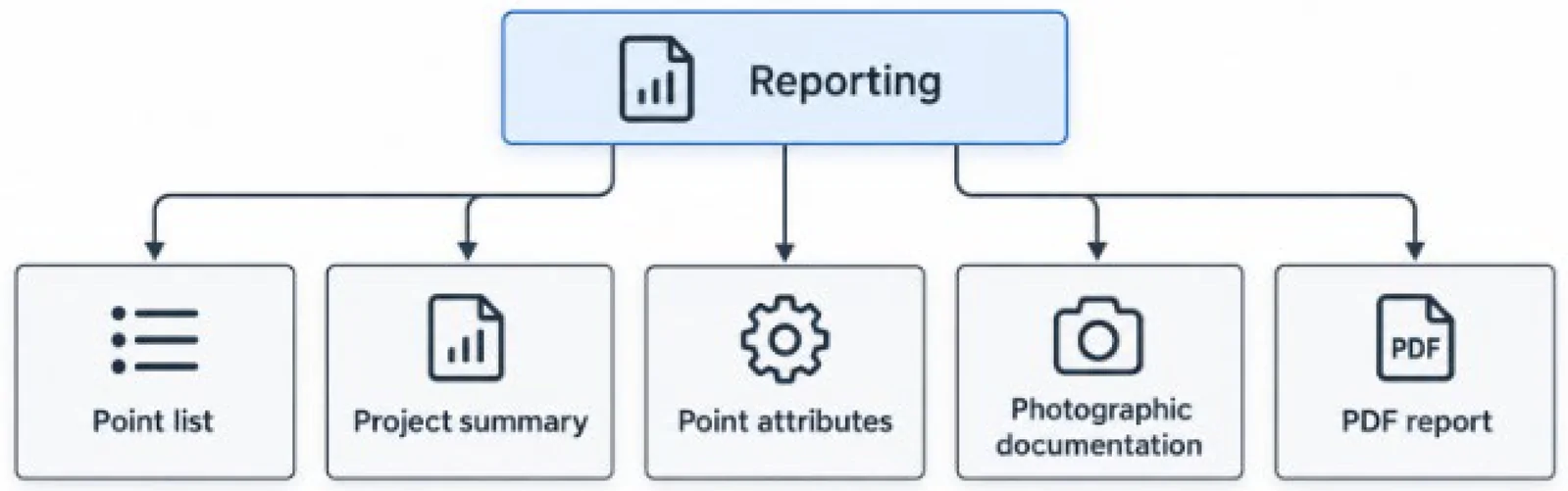
Reporting module of the “Do Punktu” application.

The final major functional module is the reporting module (Figure 7). The application enables the generation of PDF reports containing a list of points and selected descriptive information. A report may serve as a supplementary document after the completion of fieldwork, support data archiving, or facilitate the transfer of information to other project participants. The reporting function completes the fieldwork cycle involving geodetic points. The user can prepare a project, import or add points, locate them in the field, supplement their documentation, and then generate a summary for all points or for points selected according to specific attributes—for example, only damaged or unlocated points. Consequently, the application is not limited to a single task but supports the complete workflow associated with geodetic point management.

## 5. Field Testing of the Application

The research presented in this paper primarily covers the design, implementation, and functional testing of the “Do Punktu” application. Due to the limited scope of the study, a comprehensive metrological validation of smartphone positioning accuracy was not conducted. The GNSS observations described later in this section were preliminary and were intended to assess the practical usefulness of the application’s navigation functions. A detailed analysis of smartphone-based positioning accuracy—including the measurement methodology, comparison of devices, observation conditions, error analysis, and the potential use of smartphones in geodetic tasks—will be addressed in a separate study.

The field tests were divided into three main areas (Table 5). At the current stage, the tests were functional in nature and did not constitute a quantitative validation of the application’s effectiveness.

### 5.1. Point Inventory

**Figure 8:**
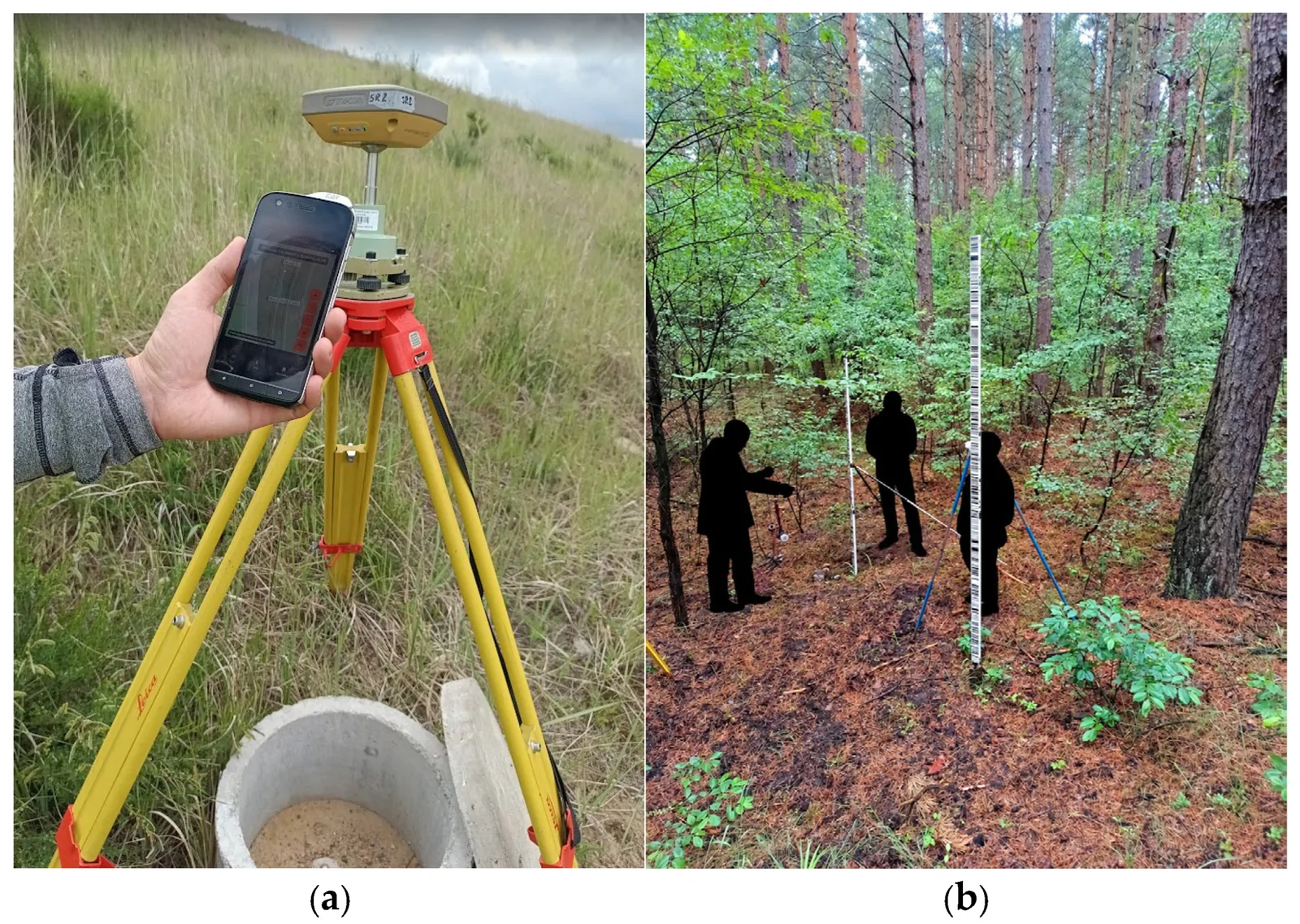
Inventory: (**a**) static GNSS measurement and navigation verification at a point; (**b**) precise levelling of reference points.

The first and most time-consuming stage involved the inventory of points in three test areas representing different types of surveying networks. This work was carried out by surveying teams using conventional topographic maps and point descriptions to create the database. The coordinates of the reference points were determined using static GNSS observations (Figure 8a). In forested areas, traverse measurements and precise levelling were additionally used (Figure 8b).

The primary test area was a network established for student education at AGH University of Krakow. It comprised elements of the national control network and additional points stabilized for field classes conducted at the university. The network consisted of 129 points in total. Of these, 50 were located in the field, while the remaining points were classified as destroyed.

The second test area was the mining area of the Bochnia Salt Mine. The inventory conducted as part of the project confirmed the existence of 138 points subject to measurement at four-year intervals. Most of the benchmarks were wall benchmarks installed on buildings in a low-rise urban environment. Approximately 20% of the network consisted of ground points, often located in forested or open rural areas, which significantly complicated their identification.

The third test area was an inactive KGHM flotation-tailings reservoir located in the Lower Silesian Voivodeship. The reservoir has not been operated since 1968. The inventory confirmed the existence of 272 measurement points located in forested areas and on deposited sediments. In accordance with the requirements of the mining authority, the network is surveyed annually.

### 5.2. Functional Testing

The second stage was conducted after the point databases had been created and the associated photographs and available topographic descriptions had been collected. Its purpose was to assess the intuitiveness of the implemented solutions and the usability of the application itself.

**Figure 9:**
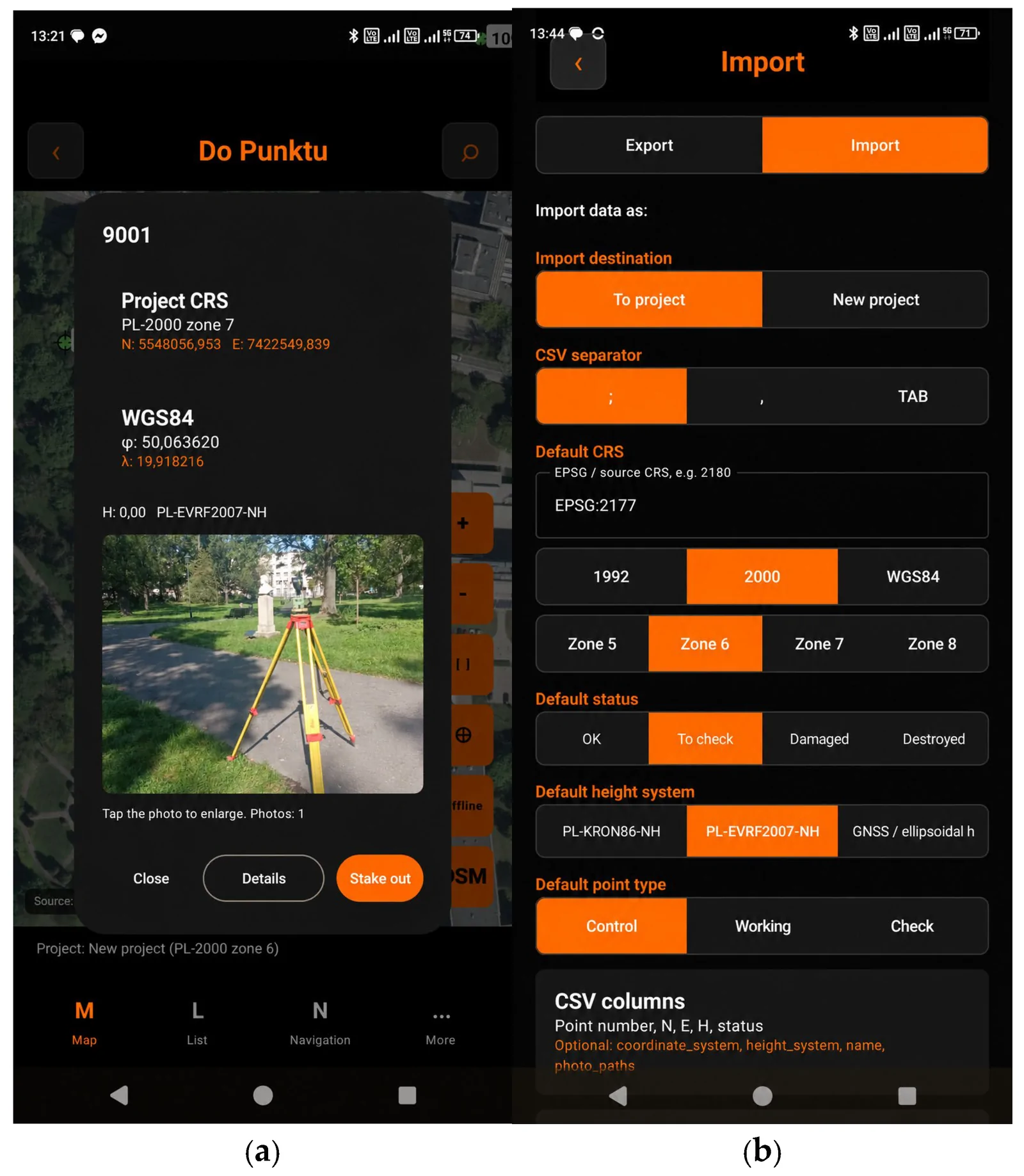
Application view: (**a**) point details preview, (**b**) data import into the project.

The testing covered:

- data-import and data-export modules (Figure 9b);
- report-generation functions;
- coordinate-transformation and correction algorithms;
- selection of the height model and height-transformation parameters;
- display of photographs and topographic descriptions (Figure 9a);
- adding data in the field;
- subsequent export and transfer of locally stored project data.

The user evaluation was conducted as formative functional and usability testing. It focused on identifying operational difficulties, collecting user feedback, and checking whether the implemented functions supported the intended field tasks. The references to ISO 9241-11 and ISO 9241-210 [18,19] provided a conceptual framework for considering usability and user-centred design. No standardized usability questionnaire or quantitative measures of task completion time, success rate, or user error rate were collected. Consequently, this stage provides qualitative evidence for further development rather than a quantitative assessment of usability or a formal demonstration of conformity with these standards.

The student testers participating in the research project identified several usability problems. These included insufficient font readability against map backgrounds. In response, the application was modified to allow users to enlarge the font and adjust the contrast. Testers also reported that pressing the smartphone’s system “Back” button caused the application to close. This behaviour was redesigned so that the button returned the user to the previous screen.

Another difficulty involved preparing a correctly formatted CSV file for data import. Consequently, a dedicated website and user-help documentation were created to explain the application’s functions and the required data formats.

The scope and results of the second stage are summarized in Table 6.

### 5.3. Numerical Verification of Coordinate Conversions

The numerical consistency of the horizontal coordinate conversions was assessed against MATLAB R2024b (Update 4) with Mapping Toolbox 24.2, using the projcrs, projinv, and projfwd functions [32]. The test comprised 30 synthetic input points: five in each of the four PL-2000 zones, five in PL-1992, and five expressed as geographic coordinates labelled WGS84 in the application. Sixty conversions were evaluated using results generated by “Do Punktu” running on an Android emulator. The maximum horizontal discrepancy was 0.153 mm, and the root-mean-square horizontal discrepancy was 0.032 mm. For geographic outputs, angular differences were converted to local linear equivalents using the GRS80 ellipsoid. The comparison followed the application’s assumption of no geographic reference-frame shift and did not include transformations between reference-frame realizations or epochs, height conversions, or an assessment of GNSS measurement accuracy. These results demonstrate numerical agreement for the tested projection calculations.

### 5.4. Field Testing of Offline Operation and GNSS Navigation

The third stage involved testing the offline operation of the application in the field. The work was conducted across all three networks in June 2026. 

The devices included Samsung Galaxy A53 5G, Galaxy A57 5G, Galaxy XCover 6 Pro, and Galaxy S25 Ultra (Samsung Electronics, Suwon, Republic of Korea); two HAMMER CONSTRUCTION 2 5G Military Edition devices (mPTech, Warsaw, Poland); Xiaomi Redmi Note 15 Pro 5G and two Redmi Note 15 devices (Xiaomi Corporation, Beijing, China); and Motorola Moto G86 5G and Moto G17 4G (Motorola Mobility, Chicago, IL, USA). The devices operated on different Android versions, depending on their model and installed software updates. As the exact operating-system versions used by individual devices during the field campaign were not systematically recorded, they are not specified separately. The participants were different from the teams involved in the inventory stage.

The tests were performed by 11 surveyors. These participants were different from the teams involved in the inventory stage. 

The scope of offline operation is summarized in Table 7. Core functions operate on locally stored project data. Basemap display and model-based height transformations require the relevant resources to be available on the device beforehand, whereas downloading new resources requires Internet connectivity. This distinction describes the implementation dependencies and does not imply that every function was independently evaluated under all offline conditions.

The application obtained location updates through the Android LocationManager API, registering for both GPS_PROVIDER and NETWORK_PROVIDER when enabled. Consequently, the positions available to the application were not restricted to GNSS-only fixes. The configured minimum update interval was 1 s for map positioning, coordinate measurement, and correction measurements, and 0.1 s for navigation and staking, with a minimum displacement of 0 m in all modes. These settings describe the application’s update requests rather than an experimentally measured GNSS receiver sampling rate. Android-reported horizontal accuracy was used for display, filtering, and weighted averaging where applicable; however, individual location updates and their reported accuracy values were not persistently logged during field testing.

**Figure 10:**
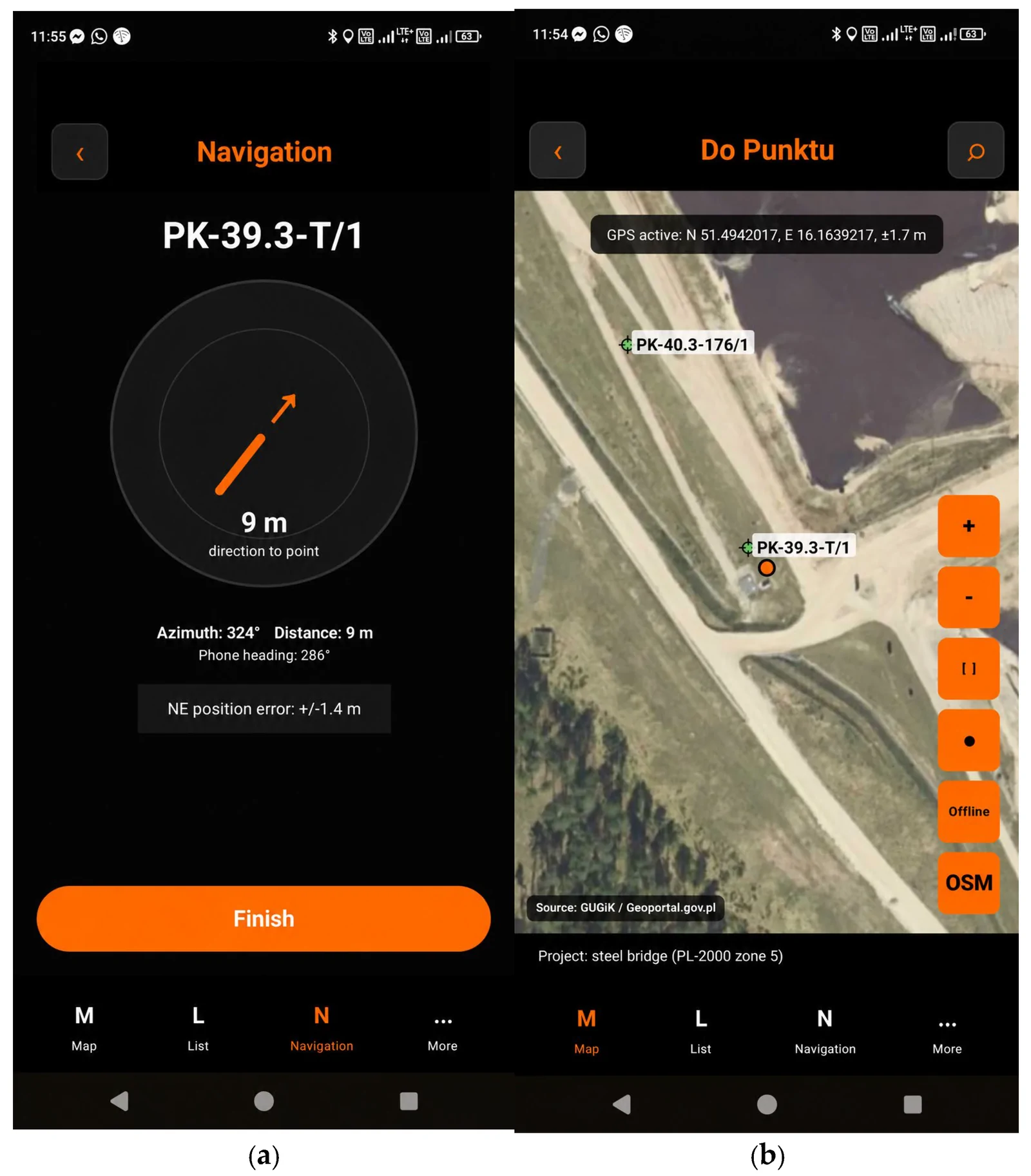
Application view: (**a**) navigation to a point, (**b**) points displayed on an orthophotomap with the user’s location.

The tests focused on the stability of navigation and the smooth operation of the application modules. One of the most important elements assessed was the operational behaviour and practical usefulness of navigation to a selected point (Figure 10).

Preliminary field observations indicated that the distance between the displayed position and the target point was approximately 4–5 m in open terrain and approximately 10 m in forested areas. These values are exploratory because no formal measurement procedure, number of independent observations, or uncertainty intervals were defined. Previous studies have examined smartphone GNSS performance under varying canopy conditions [33]. However, the exploratory navigation offsets reported here are not directly comparable with positioning errors obtained in controlled experiments.

To reduce fluctuations in the position displayed on the map, the application applies configurable smoothing and ignores location updates with an available Android-reported horizontal accuracy greater than 20 m. This filtering applies to the map display; the navigation and staking screen does not use the same accuracy threshold. The application also includes the experimental local position-correction procedure described in Section 4.6. These processing functions should not be interpreted as evidence of improved positioning accuracy without controlled validation.

The preliminary results should not be interpreted as a metrological assessment of smartphone GNSS accuracy. Rather, they indicate the operational performance of the navigation function under the tested conditions. The integrated smartphone GNSS receiver is therefore treated as an auxiliary navigation sensor rather than as a substitute for professional geodetic GNSS equipment.

A further field study involving a new group of testers is planned after all intended system functionalities have been implemented in the application. This study will provide a more comprehensive qualitative validation of the complete system and may also constitute the basis for a subsequent quantitative assessment of positioning accuracy and fieldwork efficiency.

## 6. Discussion

The “Do Punktu” application demonstrates the feasibility of integrating organizational, geodetic, and documentation functions within a single mobile tool. Unlike solutions designed primarily for browsing spatial data, the system was developed as a local fieldwork environment focused on geodetic control points.

In contrast to geoportals and public spatial-data services, “Do Punktu” is not intended to serve as a reference source of geodetic data. Instead, it functions as a fieldwork support tool that assists users in organizing their activities, documenting points, and exchanging project data [7]. Geoportals provide access to official geodetic and cartographic resources, whereas the application focuses on the user’s workflow, point documentation, and project-data management. Accordingly, these two types of solutions should be regarded as complementary rather than competitive.

Compared with universal GIS applications such as QField, “Do Punktu” offers a narrower but more specialized scope [4,9]. It was not designed as a general GIS platform for managing arbitrary spatial layers, but as a tool dedicated to work with geodetic points. This specialization made it possible to simplify the user interface and focus the functionality on tasks typical of a surveyor’s fieldwork, including the management of points, point statuses, height information, photographs, reports, and navigation to selected points.

The closest thematically related reference in the literature is the Metrica application, described as a system supporting the collection of information about geodetic control points and navigation to those points [8,14,15]. In the case of “Do Punktu,” however, the emphasis was placed on integrating local fieldwork functions within a single mobile environment. These functions include project management, a local SQLite database, coordinate transformations, height-data management, data import and export, report generation, and a session-based GNSS-position correction module. Consequently, “Do Punktu” can be viewed as an extension of the concept of a mobile tool for surveyors toward a broader fieldwork platform.

At the same time, the limitations of the current version of the application must be emphasized. GNSS functions based on the smartphone’s built-in receiver are auxiliary and should not be regarded as a substitute for professional geodetic measurements. Positioning accuracy depends on observation conditions, the quality of the receiver integrated into the mobile device, horizon obstruction, signal multipath, and the availability of satellite systems [26,34]. Therefore, a comprehensive assessment of the GNSS modules requires separate dedicated field tests.

Another limitation of the current version is the lack of full data synchronization between users and the absence of a central database containing regularly updated point information. The application was designed primarily as a local tool. This approach increases the independence and reliability of fieldwork, particularly in areas with limited connectivity, but it restricts the ability to share documentation results among multiple users. Extending the system with data synchronization and support for collaborative work may therefore constitute one of the most important directions for future development.

The article is project- and implementation-oriented, with a focus on the application’s architecture and functionality. Further research should include field validation involving comparisons with alternative work methods, analysis of the time required to locate points, and assessment of GNSS-positioning accuracy. Despite the limitations identified above, “Do Punktu” demonstrates the feasibility of developing a specialized mobile tool that combines geodetic, documentation, and organizational functions.

At the same time, the article indicates potential development directions for commercial GIS and geodetic software developers and highlights the actual needs of professional users. In particular, the results suggest that field-oriented applications should combine specialized geodetic workflows, reliable local data storage, offline operation, structured documentation, and flexible integration with external positioning equipment.

## 7. Future Development Directions

Further development should combine technical extensions with quantitative validation of the implemented functions. The main directions are summarized in Table 8. Priority should be given to controlled positioning and correction experiments, comparative fieldwork evaluation, and quantitative usability testing.

## 8. Conclusions

This paper presented the design and implementation of the “Do Punktu” mobile application, developed as a tool supporting field geodetic work involving control points and other points used in surveying projects. The application integrates project management, a local point database, basemap support, photographic documentation, data import and export, reporting, and the auxiliary use of GNSS positioning within a single environment.

The adopted architecture, based on a local SQLite database, enables data to be stored directly on the mobile device. This capability is particularly important in field conditions, especially when Internet access is limited [21]. At the same time, the use of data-exchange formats such as CSV, JSON, KML, and ZIP enables data to be transferred between the application and other tools used in geodetic practice.

An important feature of the application is the inclusion of functions specific to geodesy, including support for the WGS84, PL-1992, and PL-2000 coordinate reference systems; selected vertical reference systems; height models; point-status documentation; and navigation and stakeout functions [25,30]. Consequently, the application is not limited to displaying points on a map but supports a broader fieldwork process.

Compared with general-purpose mapping applications and mobile GIS tools, “Do Punktu” has a more specialized character. It was designed for working with geodetic points rather than as a universal GIS system. In relation to earlier solutions such as Metrica, the application further develops the concept of a mobile tool for surveyors by transforming it into a local field platform that combines organizational, geodetic, and documentation functions.

It should be emphasized that the current version of the application is a research-and-development prototype. The GNSS functions based on the receiver integrated into a smartphone are auxiliary in nature and require further validation [34]. The assessment of positioning accuracy, the effectiveness of locating points, and the influence of the application on fieldwork duration should be addressed in a subsequent stage of research.

The presented application provides a foundation for the further development of specialized mobile tools for geodesy, both for the scientific community and for commercial companies. The principal directions for future work include:

- integration with external GNSS receivers;
- validation and refinement of the experimental local position-correction procedure;
- data synchronization;
- expanded reporting functions;
- comprehensive field validation; and
- the use of the application as a research platform for evaluating the applicability of mobile devices in geodetic fieldwork.

Overall, “Do Punktu” demonstrates the feasibility of integrating organizational, spatial, positioning, and documentation functions within a single offline-first mobile environment. Although the current smartphone-based GNSS solution cannot replace professional surveying equipment, it can provide practical support for field orientation, preliminary point identification, documentation, and project-data management.

## Figures and Tables

**Table 2:** The project structure.

Model Element	Description
Project identifier	Unique project identifier in the local database
Project name	Name assigned by the user
Default coordinate reference system	Reference system used as the primary system
Project description	Additional textual information
Point list	Set of points assigned to the project

**Table 3:** The point model set of information.

Information Group	Example Fields
Identification data	Identifier, point number, name
Spatial data	Coordinate reference system, X, Y, elevation
Height data	Vertical reference system, ellipsoidal height, normal height, quasi-geoid height anomaly
Descriptive data	Description, field status
Documentation data	Paths to photographs and attachments

**Table 4:** The adopted height-data model.

Field	Meaning
Original height	Height value entered or imported by the user
Original height reference designation	Reference frame or ellipsoidal-height designation of the original value
Ellipsoidal height	Height obtained from GNSS
PL-EVRF2007-NH height	Normal height in the PL-EVRF2007-NH reference frame
PL-KRON86-NH height	Normal height in the PL-KRON86-NH reference frame
Quasi-geoid height anomaly	Interpolated model value used to convert ellipsoidal height to normal height
EVRF–Kronstadt difference	Modelled height difference between the two height reference frames

**Table 5:** Three stages of work on the application’s functionality.

Stage	Objective	Participants	Input Data	Outcome
Inventory	Creation of the point database	Surveying teams	Maps, descriptions, GNSS observations	Database containing 539 points
Functional testing	Verification of module operation	16 student testers participating in the project	Raw data and prepared projects	List of errors and improvements; website and help documentation
Field testing	Assessment of offline operation and GNSS navigation	11 surveyors	Test points	Preliminary positioning observations

**Table 6:** Results of functional application testing.

Function	Test Scope	Result Before Modification	Implemented Modification	Result After Modification
CSV import	Import of point data	Errors caused by an incorrect file format	User instructions and help documentation	Improved import process
Map interface	Readability of descriptions	Insufficient contrast	Font-size and contrast adjustment	Improved readability
System navigation	Operation of the “Back” button	Application closed	Navigation to the previous screen	Corrected behaviour

**Table 7:** Internet connectivity requirements of the implemented application functions.

Function	Offline Availability	Conditions or Limitations
Project and point management	Fully offline	Creating, viewing, editing, and deleting locally stored projects and points.
Photographic and descriptive documentation	Fully offline	Photographs, descriptions, point statuses, and associated files are stored locally.
Smartphone positioning, point navigation, and staking	Available offline	GPS-derived updates can be used without Internet connectivity. Availability of network-provider updates depends on device services and connectivity. Basemap availability is a separate requirement.
Experimental local position correction	Available offline	Requires reference coordinates and calibration observations; correction data are maintained in the current application session.
Horizontal coordinate transformations	Fully offline	Transformations between the supported coordinate reference systems are calculated locally.
Model-based height transformations	Offline after prior preparation	The required quasi-geoid or height-difference model must be downloaded or imported and available locally.
Basemap display	Offline after prior preparation	Limited to locally cached or downloaded tiles for the required area and zoom levels.
Import and export of point data	Fully offline for local files	Requires access to files stored on the device; accessing cloud-hosted files may require connectivity.
PDF report generation	Fully offline	Reports are generated locally from available project data.
Project backup and restoration	Fully offline for local files	Backup files can be created and restored locally; transfer through online services requires connectivity.
Downloading new map tiles or height models	Internet required	Requires access to the relevant external service; map downloads also depend on the selected tile source.

**Table 8:** Main directions for further development and validation of “Do Punktu”.

Area	Planned Development or Research
Positioning and external instruments	Integrate external GNSS receivers through Bluetooth, USB, or local network communication; support NMEA data and recording of solution-quality information.
Experimental local position correction	Compare positioning errors before and after correction at independent reference points; evaluate calibration duration, temporal stability, distance dependence, and observation conditions.
Fieldwork efficiency and usability	Compare point-location time, identification success rate, and documentation time with an existing workflow; collect task-performance measures and standardized usability feedback.
Architecture and interoperability	Further separate functional modules to facilitate testing and maintenance; evaluate cross-platform development while preserving compatible exchange formats.
Collaboration and synchronization	Develop controlled exchange and synchronization of projects, points, statuses, photographs, and documentation between users and devices.
Reporting	Extend report templates with maps, photographs, status summaries, data-source information, observation dates, operator details, and available positioning-quality indicators.

## Data Availability

The raw data supporting the conclusions of this article will be made available by the authors on request.
